# Subclinical systolic dysfunction in children with steroid-resistant nephrotic syndrome identified by speckle tracking echocardiography

**DOI:** 10.1186/s12887-025-05449-3

**Published:** 2025-02-04

**Authors:** Mohamed Hashem Mahgoob, Amr Mostafa Setouhi

**Affiliations:** Faculty of Medicine, Minia University, Minia, 61511 Egypt

**Keywords:** Nephrotic syndrome, Children, Systolic dysfunction, Steroid resistance, Speckle tracking echocardiography, Tissue doppler imaging, Global longitudinal strain

## Abstract

**Background:**

Steroid resistant nephrotic syndrome (SRNS) is a clinical phenotype of nephrotic syndrome (NS) that does not respond to steroid therapy and usually results in kidney failure. The aim of this study was to determine whether children with SRNS have subclinical left ventricular systolic dysfunction and, if so, to identify the risk factors for myocardial involvement in those children.

**Methods:**

This prospective case-control study included of 35 children with SRNS, 40 children in the healthy control group, and 40 children with NS during the initial episode as the diseased control group. Conventional echocardiography, tissue Doppler imaging (TDI), and speckle tracking echocardiography (STE) were performed on all the studied children.

**Results:**

No statistically significant difference in conventional echocardiography’s parameters were detected between the patient and control groups. TDI revealed that the E/E′ ratio was significantly greater in the SRNS group than in both the healthy and diseased control groups (*P* = 0.001). The left ventricle global longitudinal strain (LV GLS) was markedly lower in children with SRNS than in healthy controls and NS patients (the diseased controls) (*P* = 0.001). Multiple binary regression analysis for the predictors of systolic dysfunction in SRNS patients revealed that the serum albumin is the only variable that predicts systolic dysfunction in these children.

**Conclusions:**

Subclinical systolic and diastolic LV dysfunction should be screened in NS especially SRNS children.

## Introduction

Nephrotic syndrome (NS) is one of the most common chronic kidney diseases in children worldwide and in Egypt [1, 2]. Steroid-resistant nephrotic syndrome (SRNS) continues to be one of the most intractable causes of kidney failure [3]. The proportion of SRNS in children with NS shows ethnic variation, ranging from approximately 20% among European children to between 27% and 54% among Asian children with NS [4]. SRNS in children still represents one of the most difficult therapeutic challenges in pediatrics [1, 4].

NS, especially SRNS, may be associated with several risk factors associated with adverse cardiovascular outcomes in children [5]. Hyperlipidemia, which is a hallmark of NS, is considered a widely recognized cardiovascular risk factor. Protein wasting and systemic inflammatory activation during the pathogenesis of NS may lead to dysfunction and remodeling of the heart. Moreover, in pediatric patients with NS who experience acute afterload elevation, ventricular dysfunction may worsen [5–7].

Advanced echocardiographic techniques such as speckle tracking echocardiography (STE) provide superior outcomes when evaluating the systolic dysfunction of the LV. STE is a recently developed and susceptible technique that can assess subclinical systolic dysfunction by evaluating myocardial strain (the deformation of specific areas of the myocardium during the cardiac cycle). It is based on bidimensional (2D) echocardiographic technology and is not limited by Doppler analysis [8, 9]. Over the past decade, this new technique has gained significant importance in echocardiography laboratories because of its superior specificity compared with conventional echocardiography parameters such as the left ventricle ejection fraction (LVEF) [10].

While the LVEF will continue to be a fundamental metric for evaluating LV function, incorporating global strain (GLS) via STE enables detailed phenotyping and improved risk assessment and is a tool for present and future therapeutic advancements [11]. We conducted the present study to determine whether children with SRNS have left ventricular systolic dysfunction and, if so, to identify the risk factors for myocardial involvement in those children.

## Patients and methods

From March to October 2023, this prospective case control study was conducted at Minia University Children’s Hospital in the pediatric nephrology unit. The protocol for the study was conveyed in detail to the involved study patients, and written consent was obtained from their parents or legal guardians after ensuring the confidentiality of the information. The study was performed in accordance with the Helsinki Declaration, and the protocol was approved by both the Medical Ethics Committee and the Institutional Review Board of Minia University Hospital (No:748:5:2023).

### Sample size

For sample size calculation, the power analysis was performed via the F test with G power software. The criteria for significance were set at 0.05 (type I error) and a total power of 95%. The effect size was set at 0.4, and the sample size calculated was 105. Then, 15% was added to adjust for missed cases during the study, so the total sample size required was 120 divided into 3 equal groups, with 40 in each group.

### Study population

The study included children who were diagnosed with SRNS and were between the ages of 8 and 18. The clinical and laboratory criteria for NS were established per the KDIGO guidelines for nephrotic syndrome [12]. Children who had congenital or acquired heart disease, preexisting arrhythmia, or any accompanying systemic illness that could impair myocardial function were excluded.

The total number of SRNS children who fulfilled the inclusion and exclusion criteria was 55; 40 of them agreed to participate in the current study, and 5 of them were missed before the completion of laboratory data. We had two control groups. The first was the healthy control group, which included forty matched healthy children attending the general pediatric outpatient clinic to evaluate and judge echocardiographic parameters with no known renal, cardiac, or other chronic conditions in their medical history or clinical examination. A second disease control group comprising forty children who presented with NS during the initial episode was included to provide a better understanding of the precise influence of SRNS as a medical condition with different pathologies, genetic predispositions, and still unknown pathogeneses, its related complications, and the impact of its medications on LV dysfunction.

The children underwent a complete history and physical examination. We examined for the effect of high cumulative steroid dose on the ventricular dysfunction in children with SRNS. As the likelihood of any glucocorticoid-related adverse effect was significantly increased for each 1-gram increase in cumulative glucocorticoid dose [13]. According to a previous study done by Eid et al., [14] the studied children with NS were classified into 2 cohorts on the basis of their cumulative glucocorticoid dose in the last year: high cumulative glucocorticoid dose (> 1 gram per kilogram) and low cumulative glucocorticoid dose (< 1 gram per kilogram).The present study evaluated the complications associated with the disease and immunosuppressive drugs used. Obesity was diagnosed according to body mass index Z-score ≥ 95th percentile according to the Egyptian growth curves [15]. Cushingoid manifestations in the form of facial plethora, supra-clavicular fat pads, moon facies, acne, acanthosis nigricans, easy bruising, fine downy hair, violaceous striae [16]. Bone complications were evaluated either by history of previous pathological fracture, avascular necrosis of the femoral head, and or low bone mineral density (BMD), which was diagnosed as a BMD Z score less than − 2 by DEXA (dual x-ray absorptiometry) scans when clinically indicated [17]. Modified Ferriman-Gallwey score was used to evaluate hirsutism, a score more than or equal 8 is considered hirsutism [18]. Gum hyperplasia was assessed clinically as the gingival tissues show a granular appearance or a “cobble stones” aspect, the enlarged gingival tissues often cover the teeth, hindering oral hygiene practices and causing the accumulation of plaque [19]. Additionally, severe infections including respiratory infections, peritonitis, and CNS infection were encountered. The eGFR was determined via the modified Schwartz formula [20].

### Echocardiographic assessment of the cardiac structures and function

We used a SIEMENS ACUSON SC 2000 ultrasound system (Germany, Siemens) to conduct transthoracic echocardiography with a dedicated probe (4V1 probe) for all participants. All measurements conducted during the echocardiographic evaluation were in adherence with the recommendations of the American Society of Echocardiography/European Association of Cardiovascular Imaging (ASE/EACVI) guidelines [21]. The ejection fraction was calculated by estimating the LV end-diastolic volume (EDV, mL) and end-systolic volume (ESV, mL) via the Teichholz formula [22]. Left ventricular dimensions and left atrium dimensions were measured in M-mode in the long parasternal axis view, and left ventricular volume (LVEDV & LVESV) and left atrium volume were measured in the apical 4 chamber view [21]. Conventional and tissue Doppler imaging was utilized to assess LV diastolic function by calculating the LV E/E′ ratio, which is the ratio between the early transmitral filling (E) and average early diastolic mitral annular and basal septal velocities. LV E/E′ ratio ≥ 9 was considered suggestive of diastolic dysfunction [23].

### Two-dimensional speckle tracking

Speckle tracking echocardiography analysis was performed from apical views (the apical four chambers, the apical two chambers and the apical long axis). Standard grayscale 2D images were obtained at a frame rate of 50–90 frames/s with electrocardiogram gating during three consecutive cardiac cycles and a software package (velocity vector imaging VB10D, Siemens) was used for offline analysis. Aortic valve closure was first defined in the apical three-chamber view. The LV endocardium was then traced at each apical view at the end-systolic frame. A region of interest was automatically defined between the endo- and epicardial borders and manually adjusted to include the LV myocardium. GLS was then corrected for LVEDV & LVESV [24]. A LV GLS ratio ≤ -15 was considered suggestive of systolic dysfunction [25].

### Data analysis

All the data were collected, tabulated, and statistically analyzed via SPSS 26 for Windows (SPSS Inc., Chicago, IL, USA). The Shapiro-Wilk test was used to assess the normality of the data. Frequencies and relative percentages were used to represent qualitative data. As indicated, Fisher exact test and the chi-square test (χ2) were employed to calculate the variation between the qualitative variables. The mean value was used to express quantitative data, along with the standard deviation (SD). In the case of nonparametric variables, the Kruskal-Wallis test was used to determine the difference between three groups of quantitative variables, whereas the one-way ANOVA test was used for parametric variables. The variance across groups was ascertained through the implementation of post hoc assessment. Mann-Whitney U tests and independent t-tests were employed to determine the differences between the two groups, specifically for nonparametric and parametric data. Pearson and Spearman correlation analyses were conducted to ascertain the correlations between various variables. Predictive factors of LV dysfunction were subsequently identified via multivariate binary logistic regression analysis. We used forward selection to choose variables for multivariate analysis. Two-tailed tests were applied for every significant difference. The p-value threshold for significance was set to ≤ 0.05.

## Results

The clinical and baseline demographic characteristics of the studied children were described in Table 1. We enrolled 115 children who met our specified inclusion criteria, including 35 with SRNS, 40 with NS during the initial episode, and 40 healthy controls. The mean age of the children included in our research was 10.5 ± 1.7 years for the healthy controls, 11.3 ± 1.5 years for the SRNS group, and 11.1 ± 1.4 years for the NS (initial episode) group. The average duration of NS was 33.5 ± 15.5 months for SRNS patients and 1.5 ± 0.5 months for NS patients (initial episode). Prior to resistance development, the clinical course of the SRNS group included two cases of infrequent relapse (5.7%), seven cases of frequent relapse (20%), six cases of steroid-dependent NS (17.1%), and 20 cases of initial SRNS (57.2%). More elevated mean systolic and diastolic blood pressures were observed in the SRNS patients than in the diseased patients and normal controls. The SRNS group utilized steroid-sparing drugs and steroids (high cumulative glucocorticoid use) at a higher rate than the NS control group did (*P* < 0.001).

**Table 1:** Demographic and clinical data of the studied children

Variable	SRNS (*n* = 35)	NS during the initial episode (*n* = 40)	Healthy controls (*n* = 40)	*p* value
**Male/female**	20/15 ^***c***^	22/18 ^**b**^	20/20	0.05*
**Age at enrollment** (years) (mean ± SD)	11.3 ± 1.5	11.1 ± 1.4	10.5 ± 1.7	0.06
**Age at diagnosis** (years) (mean ± SD)	6.3 ± 2.5	7.1 ± 1.3		0.06
**Duration of illness** (months) (mean ± SD)	32.4 ± 7.2	1 ± 0.3		< 0.001*
**Course before developing resistance: (no%)**				
IFRNS	2 (5.7%)			
FRNS	7 (20%)			
SDNS	6 (17.1%)			
SRNS from the start	20 (57.2%)			
**Systolic Blood pressure (mmHg)** (mean ± SD)	125 ± 6.9 ^***a, c***^	105 ± 6	102 ± 6.5	< 0.001*
**Diastolic Blood Pressure (mmHg)** (mean ± SD)	78 ± 7 ^***a, c***^	65 ± 2.6	64 ± 3	< 0.001*
**Biopsy findings: (no%)**				
FSGS	16 (45.7%)			
MCD	15 (42.8%)			
MesPGN	4 (11.5%)			
**Number of hospital admissions/year** (mean ± SD)	4 ± 2	1 ± 0	-----	0.03*
**Complications**:				0.001*
Persistent hypertension	11	0		
Bone complications	2	0		
Cushingoid manifestations	5	5		
Obesity	15	11		
Hypertrichosis	15	0		
Gum Hyperplasia	2	0		
Serious infection	2	0		
**Previous/current medications**:				< 0.001*
Prednisolone duration(mon) (mean ± SD)	32.4 ± 7.2	1 ± 0.3		
CGCS high/low	28/7	0/25		
Cyclosporine (yes/no)	30/5	0		
Mycophenolate Mofetil(yes/no)	20/15	0		
Cyclophosphamide(yes/no)	5/30	0		

*Significant

^a^ indicates a significant difference between SRNS and NS during the initial episode

^b^ indicates a significant difference between NS patients during the initial episode and healthy controls

^c^ indicates a significant difference between patients with SRNS and healthy controls

**Abbreviations**: CGCS: cumulative glucocorticoids; FRNS: frequently relapsing nephrotic syndrome; FSGS: focal segmental glomerulosclerosis; IFRNS: infrequently relapsing nephrotic syndrome; MCD: minimal change disease; MesPGN: mesangioproliferative glomerulonephritis; mmHg: millimeter mercury; n: number; SD: standard deviation; SDNS: steroid-dependent nephrotic syndrome; SRNS: steroid-resistant nephrotic syndrome; SSNS: steroid-sensitive nephrotic syndrome

Table 2 showed the laboratory and echocardiographic parameters of the studied groups. Serum albumin, cholesterol, and urinary P/C ratios were significantly different between children with SRNS or NS during the initial episode and healthy controls (*P* = 0.001). The SRNS group had a significantly lower eGFR than the NS group and healthy control group (*P* = 0.001). No significant differences in conventional echocardiography parameters were detected between the control and patient groups. TDI revealed that the E/E′ ratio was significantly greater in the SRNS group than in both the healthy and diseased control groups (*P* = 0.001). LV GLS with speckle tracking echocardiography was significantly lower in children with SRNS than in those with NS during the initial episode and healthy controls (*P* = 0.001). Additionally, the LV GLS was significantly lower in NS patients than in healthy controls during the initial episode (*P* = 0.001).

**Table 2:** Laboratory and echocardiographic parameters of the studied children

Variable	SRNS (*n* = 35)	NS during the initial episode (*n* = 40)	Healthy controls (*n* = 40)	*p* value
**Serum creatinine (mg/dL)** (mean ± SD)	0.8 ± 0.2	0.7 ± 0.14	0.74 ± 0.13	0.09
**Serum urea (mg/dL)** (mean ± SD)	40.5 ± 16	35.9 ± 9.5	32.9 ± 6	0.2
**eGFR (mL/minute/1.73 m**^**2**^**)** (mean ± SD)	105 ± 9 ^***a, c***^	117 ± 7	122.6 ± 9.6	0.001*
**Serum Na (mEq/L)** (mean ± SD)	137.8 ± 3.07	138.5 ± 3.2	139.2 ± 3.9	0.16
**Serum K (mEq/L)** (mean ± SD)	3.9 ± 0.38	4.08 ± 0.27	4.09 ± 0.27	0.07
**Serum Ca** (**mg/dL)** (mean ± SD)	10 ± 0.60	9.7 ± 0.62	10.3 ± 0.72	0.08
**Serum albumin (mg/dL)** (mean ± SD)	2.6 ± 0.4^***c***^	2.7 ± 0.4^***b***^	4.3 ± 0.2	0.001*
**Serum cholesterol (mg/dL)** (mean ± SD)	325.7 ± 35^***a, c***^	259 ± 32 ^***b***^	135 ± 13.7	0.001*
**Urinary P/C ratio** (mean ± SD)	2.9 ± 0.9^***a, c***^	1.6 ± 1^***b***^	0.15 ± 0.05	0.001*
**EF (%)** (mean ± SD)	63.6 ± 2.4	63.3 ± 2.4	63.2 ± 2.4	0.6
**FS (%)** (mean ± SD)	33.9 ± 1.9	33.6 ± 1.5	34.4 ± 1.9	0.2
**LAD (mm)** (mean ± SD)	22.2 ± 4.3	23.35 ± 3.2	22.1 ± 2.2	0.3
**LV ESV (mm) (**mean ± SD)	23.2 ± 3.2	22.9 ± 2.4	24.4 ± 1.6	0.6
**LV EDV (mm)** (mean ± SD)	39 ± 5	38 ± 4	37 ± 5	0.2
**LV GLS (%)** (mean ± SD)	13.8 ± 1.7 ^***a, c***^	16.9 ± 1^***b***^	18.8 ± 1.2	0.001*
**E/E**′** ratio** (mean ± SD)	8.3 ± 1 ^***a, c***^	7.1 ± 0.5	6.9 ± 0.4	0.001*

*Significant

^a^ indicates a significant difference between SRNS and NS

^b^ indicates a significant difference between NS and healthy controls

^c^ indicates a significant difference between SRNS and healthy controls

**Abbreviations**: eGFR: estimated glomerular filtration rate; E/E′: early transmitral/early diastolic mitral annular velocity ratio; EF: ejection fraction; FS: fractional shortening; GLS: global longitudinal strain; LAD: left Atrial Diameter; LV: left ventricle; EDV: end diastolic volume; ESD: end Systolic volume; n: number; P/C ratio: protein creatinine ratio; SRNS: steroid-resistant nephrotic syndrome

Subclinical systolic dysfunction was found in 25 (71%) children with SRNS (LV GLS ratio ≤ -15). Also, diastolic dysfunction was suspected in 15 (43%) children with SRNS (E/E′ ratio ≥ 9). A comparison between SRNS children with and without systolic dysfunction revealed significant differences in diastolic blood pressure and serum albumin and in those receiving cyclosporine (*P* = 0.03 for all of them) (Table 3). On the other hand, when we compared children with and without diastolic dysfunction, we detected significant differences with respect to systolic and diastolic blood pressure; high CGCS; cyclosporine; mycophenolate mofetil; and serum creatinine, albumin, cholesterol, and LV GLS (Table 4).

**Table 3:** Comparison between SRNS children with and without systolic dysfunction

Variable	SRNS with impaired GLS (*n* = 25)	SRNS with normal GLS (*n* = 10)	*p* value
**Age (years)**	11.6 ± 1.5	10.8 ± 1.4	0.15
**Sex (male)**	15(60%)	5(50%)	0.17
**Duration of illness (months)** (mean ± SD)	33.2 ± 6.8	29.5 ± 8	0.06
**Course before developing resistance**			
IFRNS	1 (4%)	1 (10%)	0.74
FRNS	6 (24%)	1 (10%)	
SDNS	4 (16%)	2 (20%)	
SRNS from start	14 (56%)	6 (60%)	
**Biopsy finding**			
MCD	10(40%)	5 (50%)	0.86
FSGS	12 (48%)	4 (40%)	
MesPGN	3 (12%)	1 (10%)	
**Systolic Blood pressure (mmHg)** (mean ± SD)	127 ± 7.4	121.7 ± 2	0.1
**Diastolic Blood Pressure (mmHg)** (mean ± SD)	79 ± 7.6	74.5 ± 4.4	0.03*
**Cyclosporine (n%)**	25 (100%)	5 (50%)	0.03*
**CGCS high(n%)**	23 (92%)	5 (50%)	0.1
**Mycophenolate Mofetil(n%)**	10 (40%)	2 (20%)	0.5
**Cyclophosphamide(n%)**	2 (8%)	3 (30%)	0.09
**Serum creatinine (mg/dL)** (mean ± SD)	0.9 ± 0.2	0.8 ± 0.1	0.5
**Serum urea (mg/dL)** (mean ± SD)	38.8 ± 14.3	46 ± 21.4	0.4
**eGFR (mL/minute/1.73 m**^**2**^**)** (mean ± SD)	105 ± 9.6	105.6 ± 7.5	0.5
**Serum Na (mEq/L**) (mean ± SD)	137.6 ± 2.8	138.2 ± 3.4	0.59
**Serum K (mEq/L)** (mean ± SD)	3.8 ± 0.28	4 ± 0.34	0.37
**Serum Ca** (**mg/dL)** (mean ± SD)	9.8 ± 0.7	10.1 ± 0.9	0.29
**Serum albumin (mg/dL)** (mean ± SD)	2.5 ± 0.4	2.9 ± 0.3	0.03*
**Serum cholesterol (mg/dL)** (mean ± SD)	328.5 ± 36	316 ± 31.3	0.3
**Urinary P/C ratio** (mean ± SD)	3 ± 0.9	2.7 ± 1	0.3
**EF (%)** (mean ± SD)	63.4 ± 2.5	64.5 ± 2.2	0.1
**FS (%)** (mean ± SD)	54.1 ± 2.1	33.2 ± 0.7	0.1
**E/E**′** ratio** (mean ± SD)	8.4 ± 1.1	7.8 ± 0.7	0.2

*Significant

**Abbreviations**: CGCs: cumulative glucocorticoids; FRNS: frequently relapsing nephrotic syndrome; FSGS: focal segmental glomerulosclerosis; IFRNS: infrequently relapsing nephrotic syndrome; MCD: minimal change disease; MesPGN: mesangioproliferative glomerulonephritis; eGFR: estimated glomerular filtration rate; E/E′: early transmitral/early diastolic mitral annular velocity ratio; EDV: end diastolic volume; EF: ejection fraction; FS: fractional shortening; GLS: global longitudinal strain; LV: left ventricle; n: number; P/C ratio: protein creatinine ratio; SDNS: steroid-dependent nephrotic syndrome; SRNS: steroid-resistant nephrotic syndrome

**Table 4:** Comparison between SRNS children with and without diastolic dysfunction

Variable	SRNS with diastolic dysfunction (*n* = 15)	SRNS with normal diastolic function (*n* = 20)	*p* value
**Duration of illness (months)**	34.2 ± 3	1.1 ± 9	0.1
**Systolic Blood pressure (mmHg)**	130 ± 7.9	122.5 ± 3.8	0.01*
**Diastolic Blood Pressure (mmHg)**	84.2 ± 4.9	73.3 ± 4.9	0.001*
**Course before developing resistance**			
IFRNS	1 (6.7%)	1 (5%)	0.95
FRNS	3 (20%)	4 (20%)	
SDNS	2 (13.3%)	4 (20%)	
SRNS from start	9 (60%)	11 (55%)	
**Biopsy finding**			
MCD	6 (40%)	9 (45%)	0.93
FSGS	7 (46.7%)	9 (45%)	
MesPGN	2 (13.3%)	2 (10%)	
**Cyclosporine (no%)**	15(100%)	15(75%)	0.03*
**CGCS high (no%)**	15(100%)	13(65%)	0.01*
**Mycophenolate Mofetil (no%)**	10(66.7%)	2(10%)	0.001*
**Cyclophosphamide (no%)**	0(0%)	5(25%)	0.03*
**Serum creatinine (mg/dL)**	1 ± 0.17	0.8 ± 0.19	0. 09
**Serum urea (mg/dL)**	37.3 ± 9	43 ± 19.8	0.8
**eGFR (mL/minute/1.73 m2)**	105.9 ± 11.5	104.6 ± 6.9	0.6
**Serum Na (mEq/L)** (mean ± SD)	137.4 ± 2.9	138.1 ± 3.1	0.50
**Serum K (mEq/L)** (mean ± SD)	3.7 ± 0.35	3.9 ± 0.32	0.08
**Serum Ca** (**mg/dL)** (mean ± SD)	9.9 ± 0.6	10.1 ± 0.5	0.29
**Serum albumin (gm/dL)**	2.3 ± 0.4	2.8 ± 0.4	0.002*
**Serum cholesterol (mg/dL)**	347 ± 27.8	309.7 ± 31.7	0.001*
**Urinary P/C ratio**	3 ± 1	2.8 ± 0.9	0.6
**EF (%)**	73.2 ± 2.5	74 ± 2.3	0.1
**FS (%)**	44.2 ± 2.1	43.6 ± 1.8	0.2
**EDV (ml)**	95 ± 5.7	93.6 ± 5.1	0.06
**LV GLS (%)**	13 ± 1.3	14.4 ± 1.7	0.01*

*= Significant (*p* < 0.05)

**Abbreviations**: CGCs: cumulative glucocorticoids; FRNS: frequently relapsing nephrotic syndrome; FSGS: focal segmental glomerulosclerosis; IFRNS: infrequently relapsing nephrotic syndrome; MCD: minimal change disease; MesPGN: mesangioproliferative glomerulonephritis; eGFR: estimated glomerular filtration rate; E/E′: early transmitral/early diastolic mitral annular velocity ratio; EDV: end diastolic volume; EF: ejection fraction; FS: fractional shortening; GLS: global longitudinal strain; LV: left ventricle; n: number; P/C ratio: protein creatinine ratio. SDNS: steroid-dependent nephrotic syndrome; SRNS: steroid-resistant nephrotic syndrome

LV GLS was significantly negatively correlated with the duration of illness; systolic and diastolic blood pressure; and the use of cyclosporine, serum albumin, serum cholesterol, and the E/E′ ratio (Table 5). Table 5 showed positive correlations between the E/E′ ratio and both the systolic and diastolic blood pressure; high CGCS; cyclosporine; mycophenolate mofetil; and cyclophosphamide, serum creatinine, serum albumin, serum cholesterol, EF, and LV GLS.

**Table 5:** Correlations between left ventricular GLS, the E/E′ ratio and other variables among children with SRNS

Variable	LV GLS %		E/E′ ratio	
	*r*	*p*	*r*	*p*
**Duration of illness (months)**	-0.39	0.01*	0.1	0.5
**Systolic Blood pressure (mmHg)**	-0.47	0.004*	0.51	0.002*
**Diastolic Blood Pressure (mmHg)**	-0.48	0.003*	0.82	0.001*
**CGCS high**	-0.26	0.08	0.30	0.03*
**Cyclosporine**	-0.3	0.04*	0.46	0.001*
**Mycophenolate Mofetil**	-0.21	0.17	0.5	0.001*
**Cyclophosphamide**	0.26	0.08	-0.30	0.03*
**Serum creatinine (mg/dL)**	-0.23	0.17	0.61	0.001*
**Serum urea (mg/dL)**	0.26	0.12	0.17	0.3
**Serum Na (mEq/L**)	0.9	0.5	-0.07	0.65
**Serum K (mEq/L**)	0.10	0.49	-0.09	0.5
**Serum Ca (mg/dL)**	0.05	0.7	-0.13	0.45
**Serum albumin (mg/dL)**	0.33	0.03*	-0.44	0.007*
**Serum cholesterol (mg/dL)**	-0.36	0.02*	0.54	0.001*
**Urinary P/C ratio**	-0.12	0.48	0.17	0.32
**eGFR (mL/minute/1.73 m** ^**2**^ **)**	-0.9	0.5	-0.04	0.8
**LV EF (%)**	0.22	0.2	-0.41	0.01*
**LV FS (%)**	0.05	0.7	-0.04	0.8
**EDV (ml)**	-0.18	0.27	0.62	0.09
**LV GLS (%)**	1		-0.40	0.01*
**E/E**′** ratio**	− 0.040	0.01*	1	

*Significant (*p* < 0.05)

**Abbreviations**: CGCs: cumulative glucocorticoids; eGFR: estimated glomerular filtration rate; E/E′: early transmitral/early diastolic mitral annular velocity ratio; EDV: end diastolic volume; EF: ejection fraction; FS: fractional shortening; GLS: global longitudinal strain; LV: left ventricle; n: number; P/C ratio: protein creatinine ratio; SRNS: steroid-resistant nephrotic syndrome

Multivariate binary logistic regression analysis for the predictors of systolic dysfunction in SRNS patients revealed that serum albumin was the only variable that predicts systolic dysfunction in these children, while systolic and diastolic blood pressure were the main predictors of diastolic dysfunction in those children (Table 6).

**Table 6:** Multivariate binary logistic regression analysis for the predictors of systolic and diastolic dysfunction in SRNS

Variables	Systolic dysfunction		
	OR	95% CI	*p*-value
**Cyclosporine**	10.7	0.96–120	0.054
**Serum albumin**	0.10	0.01–0.91	0.04*
	**Diastolic dysfunction**		
**Systolic blood pressure**	1.4	1.02-2	0.03*
**Diastolic blood pressure**	1.65	1.19–2.4	0.01*
**Serum Cholesterol**	1.05	0.99–1.11	0.07
**Serum albumin**	0.01	0-1.5	0.07

*= Significant (*p* < 0.05)

Systolic dysfunction was defined as LV GLS ≤ -15. Diastolic dysfunction was defined as LV E/E′ ratio ≥ 9

**Abbreviations**: CI: confidence interval; OR: odd ratio; SRNS: steroid-resistant nephrotic syndrome

## Discussion

Myocardial injury in patients with SRNS is an understudied field of research, especially in pediatric patients. The current research measures myocardial involvement in a manner that is by far the initial of its kind. GLS is more sensitive than LVEF for evaluating LV systolic function and provides additional prognostic information, according to previous studies over the past decade [25].

The main finding of the present study is the systolic and diastolic dysfunction in children with SRNS. There was a significant decrease in the LV myocardial deformation parameter GLS in the SRNS group compared with the NS (diseased control) group and healthy control group. Additionally, the E/E′ ratio was significantly greater in the SRNS group than in both the healthy and diseased control groups, indicating LV diastolic dysfunction. Systolic dysfunction was found in 25 (71%) children with SRNS (LV GLS ratio ≤ 15), and diastolic dysfunction was present in 15 (43%) children with SRNS (E/E′ ratio ≥ 9), which indicates subtle ventricular dysfunction in those children.

To the best of our knowledge, this is the first study to report the use of speckle tracking echocardiography for the early detection of systolic dysfunction in patients with SRNS. As SRNS is one of the most prevalent causes of CKD in children and 36-50% will develop kidney failure within 10 years [26], our findings can be compared with GLS in CKD children in a previous study by Demetgul et al. [27], who examined 38 children who had been diagnosed with CKD and revealed that GLS was lower than that of normal controls (*p* < 0.05).

LV GLS in the studied NS children during the initial episode was significantly lower than that in healthy controls, which may indicate early LV systolic dysfunction in any NS patient, even in those with recent-onset NS. This finding is in accordance with a previous report by AbdelMassih et al. [28], who reported significantly reduced GLS in NS children during the initial episode compared with that in normal controls.

Numerous hypotheses have been proposed to explain the presence of left ventricular dysfunction in NS children. Children who experience sustained proteinuria, hypoalbuminemia, thromboembolism, and exposure to steroids and cytotoxic drugs such as calcineurin inhibitors face various risk factors linked to impaired endothelial function and unfavorable cardiovascular outcomes. Additionally, they are susceptible to high oxidative stress and frequent infections. These factors, along with hyperlipidemia, are hallmark features of NS and recognized risk factors for coronary artery disease. While hyperlipidemia is usually intermittent and transient in SSNS patients, dyslipidemia and other abnormalities may persist in patients with FRNS or SRNS [6]. Many children with SRNS progress toward CKD, which is known to increase cardiovascular mortality 500- to 1000-fold compared with the general population of young adults [5]. In addition, endothelial activation attributed to an undefined “permeability factor” of extrarenal origin has long been considered a biologically plausible explanation for extrarenal endothelial alterations in NS. The recruitment of inflammatory interstitial cells and their influence on endothelial cells with extensive vascular consequences are mediated by selectin family adhesion molecules, platelet-activating factors, and the nitric oxide (NO) pathway [29].

Although the significant ventricular dysfunction in the present study detected via STE and TDI, there was no significant differences between the control and patient groups concerning the attributes of conventional echocardiographic parameters. This finding is consistent with data previously reported by Saleh et al. [30], who reported no statistically significant differences in conventional echo parameters between the control group and the SSNS and SRNS groups.

We examined the possible effects of the studied variables on ventricular dysfunction in children with SRNS. When we compared SRNS children with and without systolic dysfunction, there were significant differences in diastolic blood pressure, serum albumin, and receiving cyclosporine. Multivariate binary logistic regression analysis for the predictors of systolic dysfunction in SRNS patients revealed that serum albumin was the only variable that predicts systolic dysfunction in these children.

Since an accidental observation in 1989, serum albumin has been a consistent predictor of CVD [31]. Multiple factors may influence the association between hypoalbuminemia and adverse outcomes in cardiovascular diseases. The critical role of albumin in the maintenance of vascular integrity, vasodilatation effects, toxin binding ability, anticoagulant effects, and antioxidant capacities. Furthermore, it has been suggested that serum albumin improves the movement of cholesterol between multiple cholesterol pools, facilitating the restoration of steady-state levels as cholesterol is metabolized. Additionally, inflammatory diseases may also contribute to decreased serum albumin concentrations [32]. Endothelial dysfunction was identified in patients with NS by and has the potential to induce coronary hypoperfusion, which in turn can result in myocardial damage and, consequently, dysfunction of both the systolic and diastolic branches [28, 29].

Systolic and diastolic blood pressure in the present study significantly affected systolic and diastolic ventricular function, and they are the main predictors of diastolic dysfunction. Similarly, ***Nikorowitsch***et al. [33]. reported that elevated systolic blood pressure and elevated pulse pressure are associated with declining diastolic function, as indicated by elevated E/E′ and atrial enlargement and explained this finding by disturbed ventricular‒arterial coupling: high diastolic blood is associated with arterial stiffness, which leads to a reduction in coronary perfusion pressure, inducing myocardial ischemia and deteriorating left ventricular systolic function. It is worth noting that SRNS children in the current study had substantially greater mean systolic and diastolic blood pressures than did those in the NS and normal control groups. This finding is consistent with prior research on cardiovascular outcomes in proteinuric glomerulopathies [34]. This may be attributed to prolonged steroid ± cytotoxic therapy, as well as an increased vulnerability to chronic renal failure.

Analysis of the effects of steroid on ventricular dysfunction revealed significant effect of high CGCs on diastolic function. Glucocorticoids can increase cardiovascular risk through direct and indirect metabolic syndrome enhancement and mineralocorticoid effects, including cellular membrane electrolyte-mediated efflux. Pujades-Rodriguez et al., [35] observed an increased risk of CVDs associated with glucocorticoid dose intake even at lower doses (< 5 mg) in 6 immune-mediated diseases. The effect of steroids on diastolic dysfunction was agreed upon ***(Bispo and associates)***, who evaluated myocardial injury in a case report of a patient receiving anabolic steroids for 10 weeks [36]. Additionally, Bauer and colleagues reported that steroid intake for 8 weeks at a dosage of 5 mg/kg/day exacerbates myocardial dysfunction in mouse models [37].

On the other hand, evaluating steroid sparing agents effect on ventricular function showed the negative effect of cyclosporin; the main drug used in SRNS on both systolic and diastolic function. Multiple mechanisms through which immunosuppressive agents can impact the heart. Calcineurin inhibitors may cause cardiac hypertrophy, blood pressure elevation, dyslipidemia, and vascular remodeling [38, 39].

Notably, in the present study, the eGFR was lower in the SRNS group than in the initial episode NS and healthy control groups. Despite the inaccuracies in eGFR estimation in the presence of NS, kidney function at the time of diagnosis is a predictor of long-term risk for kidney failure. ***Popa et al.***,*** 2022*** [40] studied 125 participants who were diagnosed with NS for 7 years, at the end of their study, 34% of participants with SRNS progressed to kidney failure with either dialysis or renal transplant requirement.

The clinical course of the SRNS group included 20 (57.2%) cases were SRNS from the start, the remaining 15 cases were SDNS, FRNS, and IFRNS. Histological finding of the studied SRNS children showed that focal segmental glomerulosclerosis, minimal change disease are the main biopsy finding. This agreed with Egyptian data [41, 42] which revealed that focal segmental glomerulosclerosis, minimal change disease, and mesangioproliferative were the main histological finding in SRNS Egyptian children.

### Limitations

The present study was cross sectional, single center study, with relatively small sample size, this may limit the generalizability of the findings to broader populations.

## Conclusion

Our results revealed significant systolic and diastolic LV dysfunction in the SRNS group compared with both the disease control (children with NS during the initial episode) and healthy control groups. Based on the findings of the present investigation, early, imperceptible systolic dysfunctions of the LV can be identified via speckle-tracking analysis before their detection by conventional echocardiography. The timely identification of ventricular dysfunction is critical to prompt commencement of treatment and potentially prevent the progression of cardiovascular disease to heart failure. To validate the clinical significance of these findings, larger, longer-term studies are needed.

### Recommendations

Large confirmatory studies to shed light on the role of speckle tracking echocardiography in the diagnosis of ventricular dysfunction in children with SRNS with more longitudinal studies to use it in the clinical practice.

## Data Availability

The datasets used and/or analyzed during the current study are available from the corresponding author on reasonable request.

## Abbreviations

CGCs: Cumulative glucocorticoids
CKD: Chronic kidney disease
E/E′: Early transmitral/early diastolic mitral annular velocity ratio
EDV: End diastolic volume
EF: Ejection fraction
eGFR: estimated glomerular filtration rate
ESV: Left Ventricular end systolic volume
FS: Fractional shortening
FSGS: Focal segmental glomerulosclerosis
GLS: Global longitudinal strain
INS: Idiopathic NS
KDIGO: Kidney Disease: Improving Global Outcomes
LAD: Left atrial diameter
LV GLS: Left ventricle global longitudinal strain
LV: Left ventricle
NS: Nephrotic syndrome
P/C ratio: Protein creatinine ratio
SDNS: Steroid-dependent NS
SRNS: Steroid-resistant nephrotic syndrome
SSNS: Steroid-sensitive nephrotic syndrome
STE: Speckle tracking echocardiography
TDI: Tissue Doppler imaging
